# Distance of the radial nerve from distal interlocking screws in long-stem reverse shoulder arthroplasty: a cadaveric analysis

**DOI:** 10.1016/j.jseint.2025.03.013

**Published:** 2025-04-09

**Authors:** Ethan R. Harris, Steve S. Li, Seyedeh Zahra Mousavi, Prasenjit Saha, Mark A. Haft, Miriam D. Weisberg-Tannenbaum, Umasuthan Srikumaran

**Affiliations:** Division of Shoulder Surgery, Department of Orthopaedic Surgery, The Johns Hopkins University School of Medicine, Baltimore, MD, USA

**Keywords:** Cadaveric study, Humeral interlocking screws, Long-stem shoulder prosthesis, Periprosthetic fracture, Radial nerve, Retroversion, Reverse shoulder arthroplasty

## Abstract

Long-stem reverse total shoulder arthroplasty (rTSA) with interlocking screws is indicated for trauma and revision shoulder arthroplasty and enables fixation of the prosthesis without cement or a plate while maintaining rotational stability. We investigated the distance of the radial nerve from the distal interlocking screws of the long-stem rTSA to recommend a safe approach in placement of this prosthesis. The 3 distal 4.5-mm cortical interlocking screws of a 200-mm long-stem rTSA (FX Shoulder) were inserted with the humeral prosthesis at 0° and 20° of retroversion in 8 cadaveric specimens. A curvilinear incision from the anterolateral cubital fossa extending superolaterally along the humerus was made to expose the radial nerve between the brachialis and brachioradialis. The shortest distance from each screw to the radial nerve at 90° of elbow flexion was measured, and mean and standard deviations were calculated. At 0° of retroversion of the humeral prosthesis, the most distal 3 screws were a mean 9.5, 4.2, and 0.93 mm away from the radial nerve, respectively, whereas 20° of retroversion yielded mean screw-to-radial-nerve distances of 20, 15, and 8.7 mm, respectively. The mean distance to the radial nerve increased with increasing retroversion of the stem. We recommend using at least 20° of retroversion and making an incision anteriorly with careful retraction of soft tissues posteriorly on the humeral shaft to enable direct visualization during placement of the most distal interlocking screws to protect neurovascular structures.

The risk for iatrogenic injury to the radial nerve (RN) during distal screw placement in long-stem reverse shoulder arthroplasty (rTSA) is under-researched, despite growing use of rTSA for traumatic fractures of the humerus and revision shoulder arthroplasty. The annual volume of TSA has grown rapidly in recent years, as has the rate of periprosthetic humeral fractures.^10^ rTSA is commonly used to treat degenerative shoulder conditions, periprosthetic humeral fractures, complex humeral trauma, and for revision shoulder arthroplasty. Humeral components can be stabilized with cement, press-fit fixation, or less commonly, distal interlocking screws. Distal interlocking screw fixation of the humeral component in rTSA is useful in cases of poor proximal humeral bone quality, metaphyseal or diaphyseal bone deficits, and other scenarios resulting in challenging fixation of humeral components.^11^, ^12^, ^13^

The design of the long-stem prosthesis uses 2 distal interlocking screws for the 180-mm stem or 3 distal interlocking screws for the 200-mm stem, providing rotational stability and preventing humeral stem migration.^12^ As the number of patients with rTSA prostheses increases, and as they live longer, the use of this prosthesis has potential application in fixation of periprosthetic humeral fractures of type B and type C as classified by Wright and Cofield.^16^^,^^21^

Studies have yet to describe the risk for iatrogenic injury to the RN resulting from distal interlocking screw placement of long-stem rTSA prostheses. However, several studies have demonstrated the risk of injury to the RN from operative exposure and fixation of the humerus about the lateral intermuscular septa, as the RN courses between the brachialis muscle and brachioradialis muscle anterior to the lateral humeral epicondyle.^3^^,^^7^^,^^8^^,^^20^

In this study, we sought to determine the risk of injury to the RN by measuring the line distances between the 3 distal-most interlocking screws of a 200-mm long-stem rTSA prosthesis (Fig. 1) to the RN in fresh-frozen cadaveric upper extremities following implantation. Our goal is to provide insight and recommendations for minimizing the risk of iatrogenic injury to the RN and to describe a safe approach to distal fixation of long-stem interlocking rTSA prostheses.

**Figure 1:**
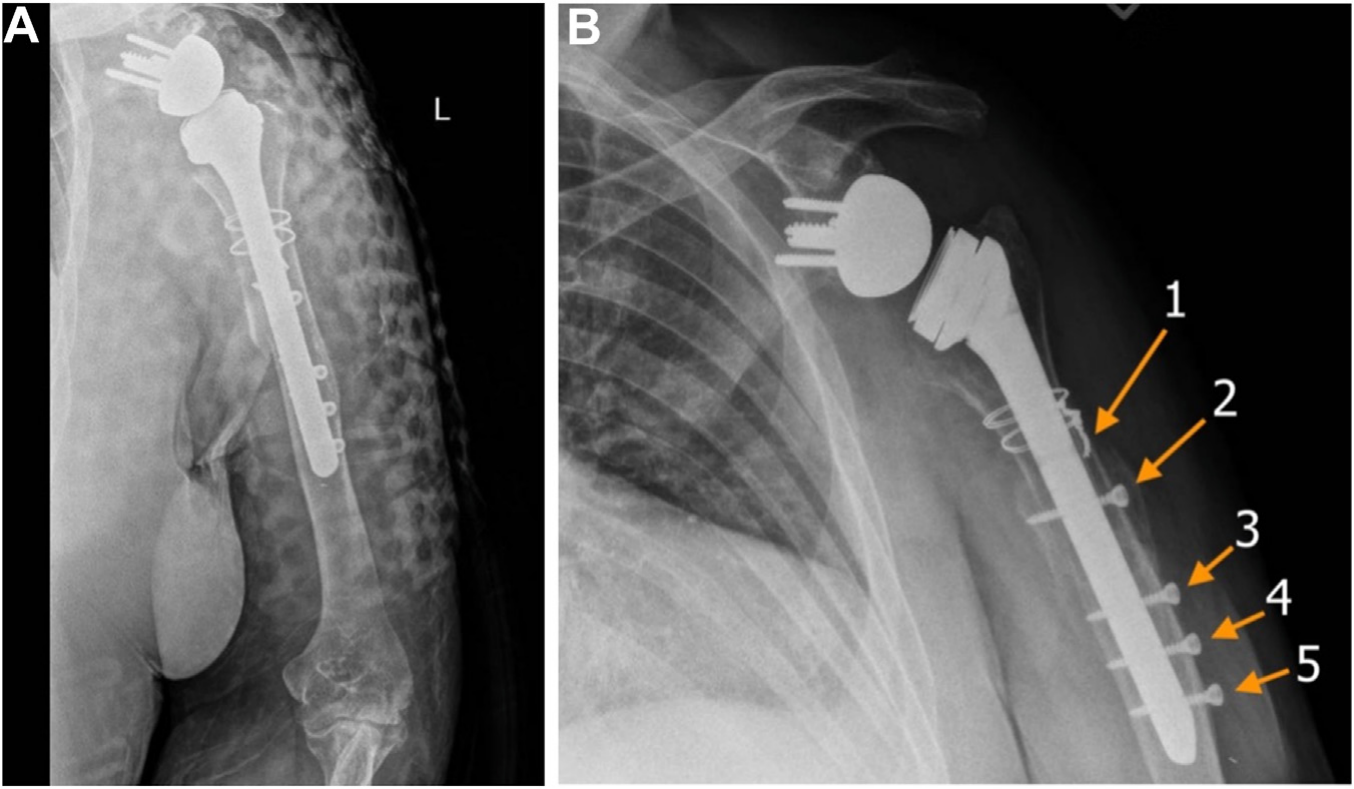
Radiographs showing a 200-mm long-stem reverse shoulder arthroplasty prosthesis with 4 interlocking screws. (**A**) Anteroposterior view taken immediately after reverse shoulder arthroplasty for fracture. (**B**) Grashey view taken 4 months postoperatively, showing interval healing. Screw hole 1 and screws 2-5 are labeled.

## Methods

**Figure 2:**
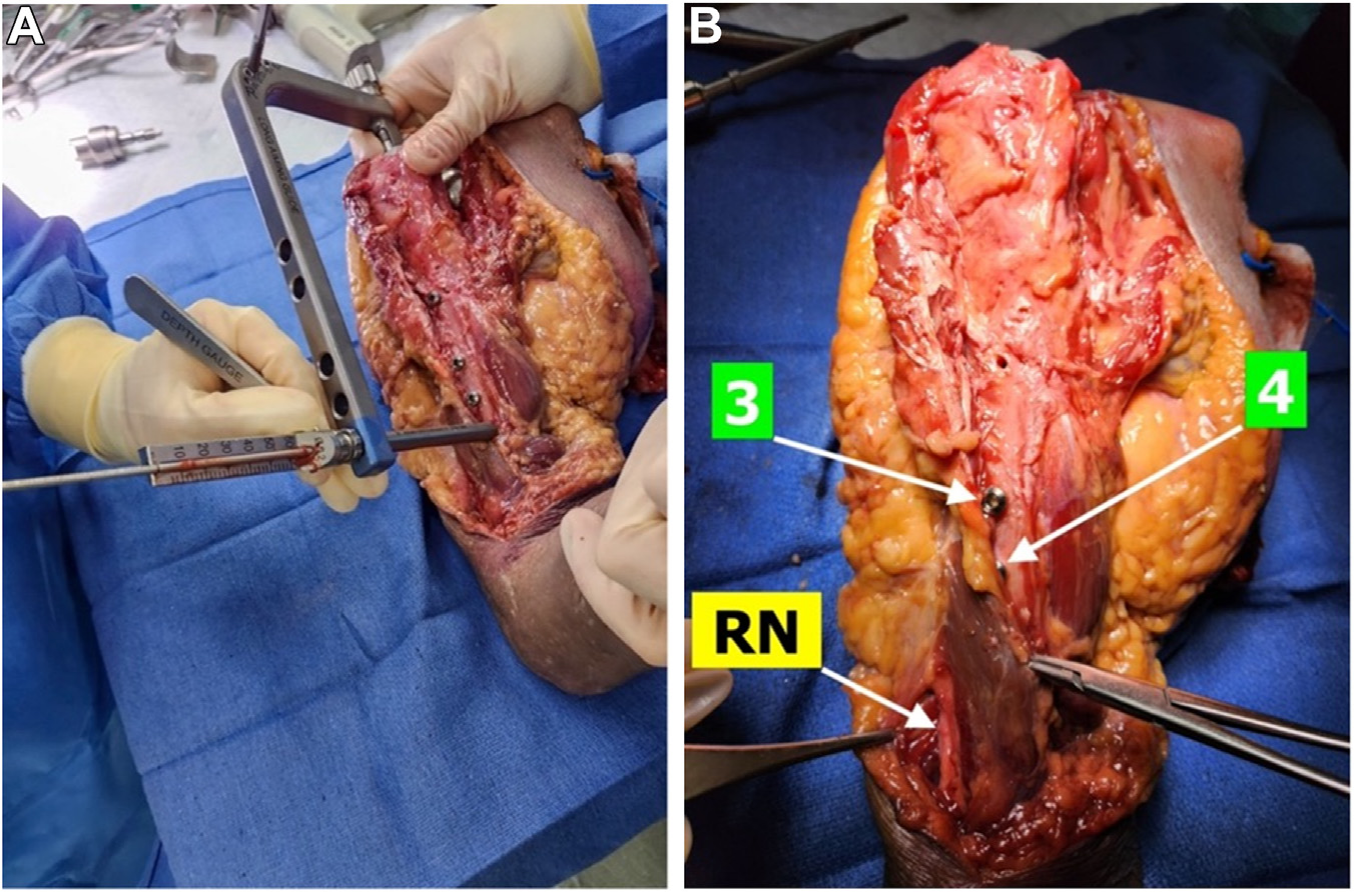
Photographs showing (**A**) drilling of the hole for screw 5 in long-stem reverse shoulder arthroplasty at 20° of retroversion; and (**B**) the radial nerve (RN) coursing between the brachioradialis and brachialis, with screws 3 and 4 labeled. Screw 5 is deep to the brachialis.

Experimental procedures were conducted on the upper extremities of 8 fresh-frozen cadaveric specimens using a 200-mm long-stem rTSA prosthesis (FX Shoulder, Dallas, TX, USA). Two specimens were male and 6 were female, with 5 right shoulders and 3 left shoulders. The specimens were thawed at room temperature before the experiments. No specimen had signs of trauma or deformity. Four specimens had undergone primary shoulder arthroplasty and 4 had undergone revision shoulder arthroplasty before donation. Three distal 4.5-mm cortical interlocking screws were used in the fixation of the humeral prosthesis at both 0° and 20° of retroversion (Fig. 2, *A*).

Exposure of the RN was performed by a fellowship-trained, board-certified orthopaedic shoulder surgeon (U.S.) with more than a decade of experience performing rTSA. A curvilinear incision was made at the anterolateral cubital fossa and extended superolaterally along the humerus. This method enabled visualization of the RN situated between the brachialis and brachioradialis muscles, ensuring minimal disruption to surrounding anatomical structures (Fig. 2, *B*), as well as extraction of the primary shoulder arthroplasty components. The 200-mm humeral prosthesis was selected over the 180-mm prosthesis because screws 3 and 4 are in the same positions for both implants. The proximity of the 3 distal-most interlocking screws (herein referred to as screws 3-5) relative to the RN were of interest because their level of insertion is close to the descent of the RN as it courses from the posterior compartment to the anterior compartment of the arm. The shortest line distance between the RN and each distal screw was measured by an orthopaedic surgery research fellow (M.H.) using a digital caliper for humeral prostheses implanted at both 0° and 20° of retroversion. The inside jaws of the digital caliper were placed from the screw edge to the closest border of the RN to determine the closest line distance for each measurement. Each measurement was taken with the elbow flexed to 90°, as several studies have shown variability in the position of the RN in different positions of elbow flexion.^4^^,^^6^^,^^18^ Microsoft Excel (version 2404; Build 17,541.20152 Click-to-Run; Microsoft Corp., Redmond, WA, USA) was used to tabulate data and calculate mean distances and standard deviations for each screw configuration at 0° and 20° of retroversion.

## Results

Eight cadaveric upper extremities underwent the experimental procedure. The average humeral length, measured from the most superior aspect of the humeral head to the lateral epicondyle, was 295 mm. Humeral prostheses implanted at 0° of retroversion showed the 3 distal interlocking screws to be closer to the RN relative to implantation at 20° of retroversion for each respective upper extremity (Table I). Mean distances between screws 3, 4, and 5 and the RN were significantly shorter when humeral prostheses were implanted at 0° of retroversion compared to distances when prostheses were implanted at 20° of retroversion (for screw 3: 9.5 ± 5.1 vs. 20 ± 3.8 mm; for screw 4: 4.2 ± 3.6 vs. 15 ± 3.9 mm; and for screw 5: 0.93 ± 1.8 vs. 8.7 ± 3.5 mm; all *P* < .01).

**Table I:** Radial nerve distance (in mm) from distal interlocking screws of the long-stem reverse shoulder arthroplasty humeral prosthesis as measured in 8 cadaveric shoulders.

Distal screw∗ by prosthesis retroversion	Mean ± SD	Range
At 0° of retroversion		
Screw 3	9.5 ± 5.1	4.3-19
Screw 4	4.2 ± 3.6	0.0-9.1
Screw 5	0.9 ± 1.8	0.0-4.8
At 20° of retroversion		
Screw 3	20 ± 3.8	16-25
Screw 4	15 ± 3.9	10-20
Screw 5	8.7 ± 3.5	2.1-14

*SD*, standard deviation.

∗ “Screws 3-5” refers to distal screws of the 200-mm prosthesis from proximal to distal.

With the humeral prosthesis at 0° of retroversion, the distance between the RN and screw 3 ranged from 4.3 to 19 mm. The RN traversed the positions of screws 4 and 5, with distances ranging from 0 mm to 9.1 and 4.8 mm away, respectively. With the humeral prosthesis at 20° of retroversion, the distances between the RN and the screws ranged as follows: for screw 3, 16-25 mm; for screw 4, 10-20 mm; and for screw 5, 2.1-14 mm.

## Discussion

Given the relatively recent introduction of distal interlocking long-stem humeral rTSA prostheses, there is a lack of research on patient outcomes and surgical considerations for their use. Interlocking humeral rTSA prostheses have been used successfully in cases wherein rotational stability and adequate lengthening were unachievable with primary fixation alone.^2^^,^^12^^,^^15^ Previous studies have suggested caution when considering the use of cement fixation of rTSA humeral components. In a systematic review comparing cemented vs. cementless humeral fixation in rTSA, Phadnis et al^13^ found that cemented stems were associated with a greater risk of infection, nerve injury, and thromboembolism. In addition, the authors found that early humeral stem migration was more frequent with the use of uncemented stems. The imperfections of press-fit fixation and cement fixation of humeral rTSA components, especially in technically challenging cases, led to the development of distal interlocking humeral components.^12^

The risk of iatrogenic RN palsy respective to distal interlocking screws used in humeral intramedullary nailing has been described. Greiner et al^5^ retrospectively investigated 203 patients undergoing intramedullary nailing with an implant allowing for distal interlocking screws placed either lateral-to-medial or anterior-to-posterior. They found that the use of lateral-to-medial interlocking screws resulted in a higher risk of iatrogenic RN palsy compared to the use of anterior-to-posterior interlocking screws. Theeuwes et al^19^ conducted a cadaveric study to define “danger zones” in the distal arm, wherein lateral fixation of distally interlocking humeral nails risks iatrogenic RN injury. They found that the risk of RN injury is highest from 48-122 mm proximally from the center of the capitellum and trochlea. Furthermore, several studies have demonstrated the marked anatomical variation of the RN.^3^^,^^7^^,^^8^^,^^20^

In this cadaveric study, we determined the average distance between the distal interlocking screws of a long-stem rTSA prosthesis and the RN and the effects of prosthesis retroversion on these distances. Distal interlocking screws were farther from the RN with greater retroversion of the humeral prosthesis. The proximity of the RN to all distal screws was less than 2 cm at both 0° and 20° of retroversion in all cadaveric specimens.

At 0° of retroversion of the humeral component, the screw placement for distal fixation was closer to the RN than it was at 20°. Given the lack of conclusive evidence regarding optimal retroversion of humeral rTSA components, orthopaedic surgeons using long-stem humeral prostheses for rTSA should consider the risks of 0° of retroversion given the likely close proximity of distal interlocking screws to the RN. Increasing distance is generated between interlocking screws and neurovascular structures with increasing retroversion.^1^^,^^9^ To our knowledge, no prior studies have investigated the risk of iatrogenic RN injury secondary to distally interlocking long-stem humeral prostheses in rTSA. Our results, in addition to previous studies demonstrating marked anatomical variability of neurovascular structures about the lateral aspect of the distal humerus, underscore the importance of distal screw placement when using this prosthesis design.^3^^,^^7^^,^^11^^,^^14^^,^^20^ Direct visualization of the humeral shaft during screw fixation is necessary to protect the surrounding neurovascular structures. We recommend an anterolateral approach, with careful posterior retraction of surrounding soft tissue for adequate identification of neurovascular structures.

Our study was limited by the small sample size, which cannot reflect the anatomic variability of neurovascular structures about the distal humerus. However, it is important to consider how this variability may indicate that there is more risk to iatrogenic injury than could be discerned in our study. This study was also limited by implant selection, as there is an alternative long-stem interlocking rTSA system that uses a single distal interlocking screw that is placed in a medial-to-lateral fashion. In addition, our use of fresh-frozen cadaveric samples is a limitation. Although such samples have been shown to be a viable option for operative training, the tissue quality is affected by the preservation process and is not an accurate representation of live tissue.^17^ These limitations were minimized by having an experienced, fellowship-trained surgeon perform the dissections. Future studies should explore potential anatomical safe zones when using this prosthesis.

## Conclusion

Our findings suggest that increasing retroversion results in greater distance between the RN and the distal interlocking screws in long-stem rTSA. We recommend direct visualization of this area to effectively protect the RN during interlocking of the distal screws when using long-stem prostheses.

## Acknowledgments

For editorial assistance, the authors thank Sandra Crump, MPH, and Rachel Walden, MS, in the Editorial Services group of the Johns Hopkins Department of Orthopaedic Surgery.

## Disclaimers

Funding: No funding was disclosed by the authors.

Conflict of interest: Dr Srikumaran serves as a board or committee member for American Academy of Orthopaedic Surgeons, American Shoulder and Elbow Surgeons, and Indian American Shoulder & Elbow Surgeons; reports stock or stock options from ROM3, Sonogen, and Tigon Medical; is a paid consultant for and receives intellectual property royalties from Fx Shoulder and Tigon Medical; receives other financial or material support from Arthrex, DePuy–a Johnson & Johnson Company, and Thieme; and has been a paid presenter or speaker for and received research support from Fx Shoulder. All the other authors, their immediate families, and any research foundations with which they are affiliated have not received any financial payments or other benefits from any commercial entity related to the subject of this article.

## References

[1] Berton A., Longo U.G., Gulotta L.V., De Salvatore S., Piergentili I., Calabrese G. (2022). Humeral and glenoid version in reverse total shoulder arthroplasty: a systematic review. J Clin Med.

[2] Boileau P., Raynier J.-L., Chelli M., Gonzalez J.-F., Galvin J.W. (2020). Reverse shoulder-allograft prosthesis composite, with or without tendon transfer, for the treatment of severe proximal humeral bone loss. J Shoulder Elbow Surg.

[3] Carlan D., Pratt J., Patterson J.M.M., Weiland A.J., Boyer M.I., Gelberman R.H. (2007). The radial nerve in the brachium: an anatomic study in human cadavers. J Hand Surg Am.

[4] Chen W.A., Luo T.D., Wigton M.D., Li Z. (2018). Anatomical factors contributing to radial nerve excursion at the brachium: a cadaveric study. J Hand Surg Am.

[5] Greiner F., Kaiser G., Kleiner A., Brugger J., Aldrian S., Windhager R. (2023). Distal locking technique affects the rate of iatrogenic radial nerve palsy in intramedullary nailing of humeral shaft fractures. Arch Orthop Trauma Surg.

[6] Hackl M., Lappen S., Burkhart K.J., Leschinger T., Scaal M., Müller L.P. (2015). Elbow positioning and joint insufflation substantially influence median and radial nerve locations. Clin Orthop Relat Res.

[7] Hannouche D., Ballis R., Raould A., Nizard R.S., Masquelet A.C. (2009). A lateral approach to the distal humerus following identification of the cutaneous branches of the radial nerve. J Bone Joint Surg Br.

[8] Hems T.E., Bhullar T.P. (1996). Interlocking nailing of humeral shaft fractures: the Oxford experience 1991 to 1994. Injury.

[9] Jassim S.S., Ernstbrunner L., Ek E.T. (2021). Does humeral component version affect range of motion and clinical outcomes in reverse total shoulder arthroplasty? A systematic review. J Clin Med.

[10] Kuhn M.Z., King J.J., Wright T.W., Farmer K.W., Levy J.C., Hao K.A. (2022). Periprosthetic humerus fractures after shoulder arthroplasty: an evaluation of available classification systems. J Shoulder Elbow Surg.

[11] Noger M., Berli M.C., Fasel J.H.D., Hoffmeyer P.J. (2007). The risk of injury to neurovascular structures from distal locking screws of the Unreamed Humeral Nail (UHN): a cadaveric study. Injury.

[12] Nourissat G., Corsia S., Srikumaran U., Sonnard A., Bargoin K., Paumier S. (2022). Use of a locking stem for reverse shoulder arthroplasty is a rare but reliable option. Int Orthop.

[13] Phadnis J., Huang T., Watts A., Krishnan J., Bain G.I. (2016). Cemented or cementless humeral fixation in reverse total shoulder arthroplasty? a systematic review. Bone Joint J.

[14] Qawasmi F., Dasari S.P., Safadi H., Yari S.S., Grindel S.I. (2023). Is the radial groove a myth? Is the radial nerve in direct contact with the posterior humerus?. Surg Radiol Anat.

[15] Rivera A.R., Cardona V. (2024). Locked stem reverse total shoulder arthroplasty for complex proximal humerus fracture in the elderly: clinical and radiological short-term results. J Shoulder Elb Arthroplast.

[16] Sanchez-Sotelo J., Athwal G.S. (2022). Periprosthetic postoperative humeral fractures after shoulder arthroplasty. J Am Acad Orthop Surg.

[17] Song Y.K., Jo D.H. (2022). Current and potential use of fresh frozen cadaver in surgical training and anatomical education. Anat Sci Educ.

[18] Suwannaphisit S., Aonsong W., Suwanno P., Chuaychoosakoon C. (2021). Location of the radial nerve along the humeral shaft between the prone and lateral decubitus positions at different elbow positions. Sci Rep.

[19] Theeuwes H.P., van der Ende B., Potters J.W., Kerver A.J., Bessems J.H.J.M., Kleinrensink G.-J. (2017). The course of the radial nerve in the distal humerus: a novel, anatomy based, radiographic assessment. PLoS One.

[20] Welle K., Prangenberg C., Hackenberg R.K., Gathen M., Dehghani F., Kabir K. (2022). Surgical anatomy of the radial nerve at the dorsal region of the humerus: a cadaveric study. J Bone Joint Surg Am.

[21] Wright T.W., Cofield R.H. (1995). Humeral fractures after shoulder arthroplasty. J Bone Joint Surg Am.

